# Patient Perspectives on Treatment Options for Older Women With Hormone Receptor–Positive Breast Cancer: A Qualitative Study

**DOI:** 10.1001/jamanetworkopen.2020.17129

**Published:** 2020-09-22

**Authors:** Ton Wang, Nicole Mott, Jacquelyn Miller, Nicholas L. Berlin, Sarah Hawley, Reshma Jagsi, Lesly A. Dossett

**Affiliations:** 1 Department of Surgery, University of Michigan, Ann Arbor; 2 Institute for Healthcare Policy and Innovation, University of Michigan, Ann Arbor; 3 University of Michigan Medical School, Ann Arbor; 4 Department of Internal Medicine, University of Michigan, Ann Arbor; 5 Department of Radiation Oncology, University of Michigan, Ann Arbor

## Abstract

**Importance:**

Women aged 70 years or older with hormone receptor–positive breast cancer have an excellent prognosis, but because of their age and comorbidities, they are at higher risk for treatment-related adverse events. Despite studies demonstrating the safety of omitting previously routine therapies, including sentinel lymph node biopsy (SLNB) and postlumpectomy radiotherapy, these treatments continue to be used at high rates. Physicians cite patient preference as one factor associated with overuse. However, little is known about how women view potential de-escalation of therapies.

**Objective:**

To evaluate older women’s preferences for SLNB and radiotherapy in the setting of guidelines recommending them or allowing for their omission.

**Design, Setting, and Participants:**

This qualitative study was performed from October 2019 to January 2020. Midwestern women aged 70 years and older who had never received a diagnosis of breast cancer were recruited online and interviewed. Guided by an interpretive description approach, interviews were analyzed to produce a thematic description. Data analysis was performed from January to March 2020.

**Exposures:**

Participants were presented with hypothetical scenarios in which they received a diagnosis of low-risk, hormone receptor–positive breast cancer and were given treatment options in accordance with current guidelines.

**Main Outcomes and Measures:**

The interviews elicited perspectives on breast cancer treatment, including surgery, SLNB, chemotherapy, and postlumpectomy radiotherapy.

**Results:**

The median (interquartile range) age of the 30 participants was 72.0 (71.0-76.5) years. Most of the women were White (26 participants [87%]), lived in metropolitan areas (29 participants [97%]), and were college educated (20 participants [67%] had a 4-year degree or higher). Overall, women expressed the belief that age-based guidelines were appropriate on the basis of decreased recurrence risk and increased frailty in older patients. However, many participants stated that these guidelines should not apply to healthy older women with a long life expectancy. Some participants struggled to understand that the basis for treatment de-escalation in older patients is a favorable, not poor, prognosis. Women who said they would undergo SLNB (12 participants [40%]) perceived the procedure as low risk and providing peace of mind. Most participants (22 participants [73%]) expressed a preference for omitting postlumpectomy radiotherapy because of the perceived risks, lack of benefit, and inconvenience.

**Conclusions and Relevance:**

Positive reframing of the excellent prognosis driving national recommendations for de-escalation may reduce breast cancer overtreatment in older women. Strategies for reducing SLNB use will likely require education on the risks vs benefits and addressing patient preferences for peace of mind. In contrast, efforts to reduce radiotherapy use may need to address clinician or organizational factors.

## Key Points

**Question:**

What are older women’s perspectives on recommendations for treatment de-escalation for low-risk, early-stage hormone receptor–positive breast cancer?

**Findings:**

In this qualitative study of 30 participants aged 70 years or older, women expressed skeptical views regarding age-based treatment guidelines, difficulty interpreting the rationale for reducing low-value care to be a favorable rather than poor prognosis, and perceived benefit of some low-value therapies for peace of mind.

**Meaning:**

Emphasizing an overall favorable prognosis and improving patient education on the risks vs benefits of adjuvant therapies may help reduce overtreatment in older women with early-stage, hormone receptor–positive breast cancer.

## Teaser

This qualitative study evaluates older women’s preferences for sentinel lymph node biopsy and radiotherapy in the setting of guidelines recommending them or allowing for their omission.

## Introduction

More than one-third of patients with newly diagnosed breast cancer are aged 70 years or older.^1^ Most of these patients receive a diagnosis at an early stage, and their cancers have the favorable characteristic of expressing estrogen and progesterone receptors (ie, they are hormone receptor [HR] positive). These tumors carry an excellent long-term prognosis, and the probability of a woman aged 70 years or older dying from breast cancer is less than 1%.^2^ At the same time, older patients are more vulnerable to the risks and toxicities of cancer treatments, placing these women at high risk of overtreatment where potential harms outweigh potential benefits.^3^

Several studies^4,5,6,7,8^ have tested the safety of omitting previously routine therapies in women aged 70 years or older with early-stage, HR-positive cancer. In 2013, the Cancer and Leukemia Group B 9343 trial^4^ found that women who underwent postlumpectomy radiotherapy with or without axillary staging experienced no additional survival benefit compared with women treated with lumpectomy and endocrine therapy alone. On the basis of these and other data and as part of the Choosing Wisely campaign, the Society of Surgical Oncology recommended against routine axillary staging with sentinel lymph node biopsy (SLNB) in clinically node-negative women aged 70 years or older with HR-positive breast cancer in 2016.^5,6,7^ Similarly, the National Comprehensive Cancer Network guidelines^8^ have allowed for the omission of postlumpectomy radiotherapy in these patients since 2004.

Despite these recommendations, both SLNB and adjuvant radiotherapy continue to be used at high rates for women who are eligible for omission. In national samples, more than 80% of women aged 70 years or older with HR-positive breast cancer underwent SLNB, and more than 65% received adjuvant radiotherapy.^1,9,10,11^ Previous assessments of barriers to de-escalation of low-value breast cancer care have focused on clinician beliefs and attitudes.^12,13^ Although clinicians cite patient preferences as a factor associated with overtreatment, evidence suggests that many older patients may actually prefer a less-aggressive treatment strategy.^14^ To broadly understand this clinical decision-making scenario from the patient’s perspective, we performed a qualitative study with women aged 70 years or older to understand patient perspectives on the omission of SLNB and radiotherapy for early-stage breast cancer and to identify strategies to facilitate evidence-based and values-concordant care for patients.

## Methods

 The study was determined to be exempt from ongoing review via the University of Michigan institutional review board. All participants verbally consented to be interviewed, and a $25 gift card was offered as an incentive. This study is reported in accordance with the Standards for Reporting Qualitative Research (SRQR) reporting guideline.^15^

We conducted semistructured phone interviews with 30 participants in the Midwest from October 2019 to January 2020. Eligible participants were women aged 70 years or older, English-speaking, and without a previous diagnosis of breast cancer. Individuals who had never received a diagnosis of breast cancer were intentionally chosen for this study to eliminate influences from prior experiences. One reason for this choice is that many women who are eligible are not offered omission of these therapies by their clinicians and, thus, are unable to comment on factors influencing their decision-making. Volunteers were recruited via the UMHealthResearch.org website. Purposive sampling was used to increase diversity with respect to age, education, and race/ethnicity.

The semistructured interview guide (see the eAppendix in the Supplement) was developed in consultation with subject and methodological experts on the basis of factors hypothesized to be important in breast cancer treatment decision-making. It was piloted with 2 women and further refined for clarity. In response to topics raised in early interviews, questions were added to iteratively explore those topics in subsequent interviews.

Briefly, participants were asked to imagine a scenario in which their doctor gave them a diagnosis of early-stage, HR-positive breast cancer and recommended surgery to remove the cancer. They were asked about surgery preferences, SLNB preferences in light of age-based guidelines, and chemotherapy preferences if they were found to have a positive lymph node. In a second scenario, participants were asked to imagine undergoing a lumpectomy for the same diagnosis. They were asked about their radiotherapy preferences in relation to the age-based guideline and hormone therapy preferences. Finally, a brief demographic survey was administered.

One surgeon-scientist trained in qualitative methods (T.W.) conducted all interviews. Interviews were audio-recorded, transcribed verbatim, and deidentified. We followed the inductive and iterative approach of interpretive description,^16,17,18^ a qualitative method in the constructivist research paradigm that interprets participants’ subjective experiences to improve understanding of clinical problems. After 10 interviews, transcripts were examined, and responses were summarized by topic to a framework matrix^19^ to facilitate data immersion and preliminary analysis. On the basis of this analysis, the overall sample size of 30 was estimated to provide more than sufficient information power given the exploratory aims of the study, the analysis strategy, and the specificity of the sample. Information power is a flexible, transparent, and auditable approach to both estimating and assessing sample size in qualitative studies. It relies on key methodological principles rather than any specific technique of analysis, and it aligns well with interpretive description.^20,21^ Transcripts were imported to MAXQDA 2020 software (VERBI Software) to support coding and further analysis. The research team developed a codebook that initially contained structural and descriptive codes deductively applied for each question that were later supplemented by inductively derived codes descriptive of factors volunteered by participants. Each interview was coded independently by 2 researchers (N.M., J.M., or N.L.B.).

Full analysis proceeded via data abstraction, case comparison, and writing memoranda on clusters of codes to develop the thematic description presented here.^22^ In regular meetings, the research team discussed alternative interpretations, researcher biases, latent themes, prevalence, outlying cases, and clinical implications of findings.^16^ Cross-tabulation and typology tables in MAXQDA were used to explore potential associations among codes and demographic variables. Data analysis was performed from January to March 2020.

## Results

### Study Participants

Thirty women were interviewed, ranging in age from 70 to 84 years, with a median (interquartile range) age of 72.0 (71.0-76.5) years. Most of the women (26 participants [87%]) were White and nearly all participants lived in metropolitan areas in the Midwest (29 participants [97%]). Most were highly educated, with 20 participants (67%) holding at least a 4-year college degree (compared with 35% of US individuals).^23^ Participant demographic characteristics and treatment preferences are presented in Table 1.

**Table 1.** Summary of Participant Demographic Characteristics and Preferences for Surgery, Sentinel Lymph Node Biopsy, Chemotherapy, and Radiotherapy

Characteristic	Participants, No. (%)
Age, y	
Mean (SD)	74.0 (4.6)
Median (interquartile range)	72.0 (71.0-76.5)
70-74	20 (67)
75-79	7 (23)
80-84	1 (3)
85-89	2 (7)
Race/ethnicity	
White (non-Hispanic)	26 (87)
African American	2 (7)
Asian (Japanese)	1 (3)
Hispanic	1 (3)
Education	
High school	2 (7)
Some college or associate degree	8 (27)
Bachelor’s degree	6 (20)
Master’s degree or graduate education	14 (47)
Geographical area^a^	
Metropolitan	29 (97)
Nonmetropolitan	1 (3)
Surgery preference	
Lumpectomy	20 (67)
Mastectomy	7 (23)
No surgery	3 (10)
Sentinel lymph node biopsy preference	
Yes	12 (40)
No	15 (50)
Unsure	3 (10)
Chemotherapy preference	
Yes	15 (50)
No	7 (23)
Unsure	8 (27)
Radiotherapy preference	
Yes	6 (20)
No	22 (73)
Unsure	2 (7)

^a^ Geographical area refers to US Census–defined area according to ZIP code.

### Views on Age-Based Guidelines

Approximately one-half of participants agreed that age-based guidelines for cancer treatment were acceptable and acknowledged the value of data to inform treatment decisions. Women commonly cited a biological basis for age-based guidelines, stating that older patients may not respond to treatments or may experience increased adverse effects due to reduced stamina. Additionally, some women acknowledged that the risks of recurrence in older individuals may be outweighed by other causes of death. Many women used a social lens to reflect on age-based guidelines, suggesting they may have pursued more-aggressive treatments when they were younger because of a desire to raise children and grandchildren, but less-aggressive treatments now reflected their personal fulfillment with major life goals.

In contrast, women who stated that age-based guidelines are unacceptable suggested that other factors, such as a person’s overall health, family history, and anticipated life span, should primarily inform treatment recommendations. Some women even suggested that age-based guidelines were discriminatory, dismissive of the value of older women, and driven by financial greed. Other participants accepted a change in treatment recommendations with age but suggested that an age cutoff of 70 years was too young and that guidelines should be further stratified by age (eg, 70 vs 80 vs 90 years old). Regardless of their views on age-based guidelines, all women strongly valued autonomy and defended the right of an individual to pursue any offered treatment regardless of age, even if they would not want a treatment themselves. Importantly, several women volunteered that age-based guidelines would support their autonomy by providing options and an impetus for patient-clinician discussions. For illustrative quotations, see Table 2.

**Table 2.** Participants’ Views and Interpretations of Age-Based Guidelines

Views and interpretations	Representative quotations
Accept age-based guidelines	
Change in physiology	“There is a difference in physiology…not just hormones, the general physiology in people as they age. I don’t think the medical researchers are making subjective decisions about things like quality of life and prognosis. They’re acting using science-based research to provide the information. Therefore, it makes sense to me. Let me put it this way. It doesn’t seem weird to me or conspiratorial that the treatment options and recommendations are different based on age especially with women.” (Participant 25)
Decreased stamina	“The things that I could be susceptible to because of my age is different than what a younger person might experience, given their age, given their physical health, lots of factors.” (Participant 1)
Competing comorbidities	“Well, if there is a recurrence maybe it would be closer to the end of my lifetime anyway and maybe that wouldn’t be the thing that killed me.” (Participant 20)
Social perspective	“I’m an older person and I wouldn’t want to probably put myself through a lot for my treatment or anything. But younger people, younger women, they have their whole life ahead of them, and so they should go ahead and have it done.” (Participant 21)
Trust in research	“Well, I’m assuming that the guidelines are based on the history of women who have breast cancer… how it has been treated and how it’s responded. If that’s what makes up the guidelines, I would trust that.” (Participant 10)
Patient autonomy	“I believe older people have often been denied the ability to make their own decisions. Doctors say that’s what you do when you find it, so you do it. Without talking with the patients. Some may not have that desire to go through that for the two years they had remaining.” (Participant 18)
Oppose age-based guidelines	
Importance of health status	“It would depend on your overall health… If you were a healthy person you would make a different decision than if you were unhealthy for any reason. A younger person I think would opt for the treatment no matter what.” (Participant 11)
Improved longevity	“I’m seeing more and more healthy people in their 70s and 80s and 90s, if people eat right and get exercise, they’re healthier than they were, and people are living longer.” (Participant 29)
Genetics	“How positive can they be, whoever they are, to say that because I’m 72, the information you learn from it, this procedure won’t be helpful… It depends on my physical condition and genetics and everything else I would think.” (Participant 14)
Need for further age stratification	“I think it’s one thing if you’re in your 70s. It’s another thing if you’re in your 80s, another if you’re in your 90s and not just sort of lump it together as over 70. I mean, being in your 70s right now is like being in your, as far as I’m concerned, over 50s.” (Participant 2)
Patient autonomy	“Well, I think it’s kind of all about equality of everybody…Each person is an individual and a person should be treated as that and be able to have the benefits of the younger, as well as the older.” (Participant 12)
Financial greed	“I think that a lot of times, insurance companies are calling the shots on a lot of this stuff, and that that’s where the research is coming from. And that they don’t want to spend the money on more testing or more procedures.” (Participant 24)
Discrimination	“I don’t get this age thing. That’s just sort of a discrimination of some sort to me…I find that slightly offensive.” (Participant 14)
Interpretation of guidelines to omit sentinel lymph node biopsy and adjuvant radiotherapy	
Concern for poor prognosis	“Well, because it doesn’t do anything for my survival rate at my age. So I would feel in my mind that I have the advanced cancer, and I more than likely would die.” (Participant 21)
High outlier belief	“It’s a personal decision as far as I’m concerned. I don’t care whether a million women have had no benefit from it, I may. And that’s enough for me.” (Participant 14)
	“I don’t like to go by statistics that are meant for everybody. I’m somewhat unique in that although I’m 77, I’m in good health.” (Participant 15)
Mistrust in interpretation of research findings	“I would ask why did it not make much difference? Is it because that age group, the 70 plus for instance, has a weaker body and cannot respond very well to chemotherapy or whatever? Or is it because, did they die of some other cause? I mean there are many things that I don’t understand about just that information.” (Participant 7)
	“I guess maybe I wouldn’t trust the study. I would trust my doctor and I wouldn’t trust the study, but I would also do some more research on it, too, on my own.” (Participant 12)

### Interpretation of Guidelines to Omit SLNB and Adjuvant Radiotherapy

Many women experienced difficulty interpreting the rationale for guidelines to omit SLNB and adjuvant radiotherapy. Although the scenarios described clinical situations with a good long-term prognosis, several women assumed that SLNB and radiotherapy were not recommended because death due to the disease was inevitable. Women who preferred to omit SLNB and radiotherapy were more likely to reference the anticipated good prognosis. They either stated that the expected outcome (with lumpectomy and endocrine therapy alone) was good enough or that receiving additional interventions would not change the prognosis.

Some women were wary of applying population-based data underlying the recommendations to their individual circumstances. For example, some participants speculated that they might be healthier than the average participant in the clinical trials. Thus, they expressed a belief that they were exceptions to these guidelines because of presumed longevity based on their good health and, therefore, might benefit from aggressive treatment. Additionally, some participants questioned the reliability of the research informing the guidelines. Illustrative quotations are provided in Table 2.

### Factors in Decision-making for SLNB and Radiotherapy

Approximately one-half of participants (12 participants [40%]) said they would proceed with SLNB despite evidence suggesting that omission is safe. These women viewed the procedure as prognostic and providing peace of mind, if negative, and as providing valuable information for other treatment decisions, if positive. Overall, participants viewed SLNB as contributing minimal additional risks, even when considering it likely would require general anesthesia, whereas lumpectomy alone often does not. Women who would omit SLNB cited a desire to minimize interventions that do not provide a survival benefit and trust in their clinicians and/or national guidelines.

In contrast, most participants stated a preference to omit postlumpectomy radiotherapy (22 participants [73%]), viewing it as one of several treatment options. Participants’ perceptions of radiotherapy varied, with many believing it to be terrifying or debilitating and others believing it would be well tolerated. These beliefs were frequently conditioned by experience with friends or family who underwent radiotherapy. Another factor was the inconvenience of daily travel to a radiotherapy center. The participants who would undergo radiotherapy expressed a desire for peace of mind by eradicating any remnants of cancer. Some women stated a preference for radiotherapy to avoid hormone therapy, which they viewed as potentially having significant adverse effects and lasting for several years as opposed to only a few weeks. For illustrative quotations, see Table 3.

**Table 3.** Factors in Decision-making for Sentinel Lymph Node Biopsy and Radiotherapy

Treatment and factors	Representative quotations
Sentinel lymph node biopsy	
Supporting factors	
Prognostic test	“I would still want to get it for my own peace of mind, and then aside from that point, whether the prognosis was good or bad, to determine if I have more treatment.” (Participant 30)
	“Well, determining whether or not the cancer has spread. I mean, that’s the important thing because you may need chemotherapy. So, I would like to know that.” (Participant 22)
Peace of mind	“I know my daughter, 10 y ago they did the lymph node test, so I was glad for that for her because then she had peace of mind that nothing had spread. That’s what I would like, peace of mind on that.” (Participant 12)
Minimal risk	“Even though it wouldn’t be recommended at my age, I think I’d want to know more information and I don’t see it as really invasive.” (Participant 23)
Opposing factors	
Lack of benefit	“It doesn’t change the outcome. You’re just having more surgery with more pain and problems.” (Participant 29)
Risk of harm	“I would say as you increase in age, there’s so many other things that are going to get you and that just seems like taking a risk to have some damage done that you didn’t need to have done.” (Participant 27)
Trust in clinician and research	“I’m pretty sure that I would be going to someone at the university that I felt very comfortable with and would trust their judgment and then if they felt that the lumpectomy was enough, that would be good enough for me to follow their advice.” (Participant 13)
Radiotherapy	
Supporting factors	
Desire to eradicate cancer	“Well, if you can knock out the cancer and prevent it from reoccurring, why would you not?” (Participant 14)
Tolerable side effects	“I know people that are healthy who had the radiation and came through it fine.” (Participant 19)
	“They just zap you and that’s it. …I really wouldn’t worry about the radiation.” (Participant 21)
Avoid hormone therapy	“Well, it’s shorter term than the pill [endocrine therapy] and then you’re done with it.” (Participant 20)
Opposing factors	
Availability of other therapies	“I think I was just looking more at the practicality of the recommendation. If you can do it with a pill, that seems like an easier way to go than radiation.” (Participant 27)
Trust in clinician and research and lack of benefit	“If the studies have been done, I don’t think there would be any benefit.” (Participant 29)
Risks and fear	“I just don’t…I don’t like it, I don’t think that’s good. I think it destroys other things in your body, and I think it makes you sick.” (Participant 9)
Inconvenience of treatment	“No way I’m going to run my life about going someplace daily for medical treatment. Yeah, that’s a major factor, I’m not going to let my life revolve around my medical condition.” (Participant 4)
Societal financial cost	“The risks are not that great, but if there’s not going to be a benefit, why go through it? Why spend the money, really? And even though it’s not my money, it’s insurance money, it’s still a question of why do it?” (Participant 10)
Difference between SLNB and radiotherapy (ie, diagnostic procedure vs additional form of treatment)	“I think it’s two different things, that one is sort of like diagnostic thing, and the other one is sort of like a preventive.” (Participant 22)
	“[Radiation] doesn’t tell me that there might be something else working in there. With the lymph node thing, my understanding is that having that procedure possibly could identify something else that’s going on, so that’s why I would go with that one.” (Participant 12)

### Influence of Older Age on Breast Cancer Treatment Preferences

A consideration specific to older women is the breadth of secondhand experience they have with breast cancer. Nearly all women had 1 or more close friends or family members who have been treated for breast cancer. These prior experiences heavily influenced preconceptions about their likely prognosis, appropriate treatment, and risks and benefits from treatment. Several women knew someone who had died from breast cancer or had advanced-stage disease. These negative experiences often generated skepticism about the likely good prognosis associated with a small, HR-positive cancer and promoted a desire for aggressive treatment. For example, some women stated a preference to receive chemotherapy because they believed it had contributed to a family member’s survival. On the other hand, some participants who preferred treatment omission were influenced by positive secondhand experiences with someone who had done well with a similar choice. Many women also referenced their historical memory and the significant advances made in breast cancer treatments during their lifetimes. Some participants perceived that contemporary treatments are safer and better tolerated by older patients and thus discounted negative secondhand experiences with surgery, SLNB, chemotherapy, or radiotherapy.

Other factors important to older women were their general health, quality of life, and particular life or family circumstances. Nearly all women believed that their overall health status should be of equal or greater weight than chronological age in influencing treatment decisions. Most participants agreed that a worse health status would sway them toward less-aggressive treatments. Some would omit adjuvant therapies because they would not want to risk compromising their current functional status that enables them to serve as a caregiver to their spouse or to not be burdensome to their children. See Table 4 for illustrative quotations.

**Table 4.** Association of Older Age With Breast Cancer Treatment Preferences

Factor	Representative quotations
Historical perspective	
Secondhand experience	“Because we go by emotions and we go by what we’ve seen and we go by what we’ve experienced and what we’ve heard. And when you get in around 70 years old, you know people in nursing homes and people who’ve been in hospice.” (Participant 17)
	“When I was younger, I had two friends that passed away from breast cancer after going through all the treatments and everything.” (Participant 21)
	“I hesitate to follow that route. My mother, and this was years ago, had breast cancer and she had…I don’t know if she had removal of lymph nodes…Something done, but use of her arm was limited and it was often swollen.” (Participant 28)
	“I have, well, through my life I’ve known people that have gone through radiation for things other than breast cancer, so it just…I don’t know. It’s not something that I would want to do for myself based on what I’ve seen and feel.” (Participant 12)
Medical advances	“I think my paternal grandmother died of the effects of radiation treatment from breast cancer but goodness knows radiation back 90 years ago was a lot cruder than now.” (Participant 6)
	“Well, I think because the radiation oncology doctors are so careful about monitoring you and how careful they are about the way they do the radiation at this point in time, that is the risks are not as great as it used to be.” (Participant 10)
Factors important to older women	
General health	“Being older, there are so many factors that you’d have to consider at the moment, overall health would be the main thing. If I were older and still healthy I would choose it. If not, I would say, ‘I don’t want to add this to my other physical, well, miseries.’” (Participant 11)
Quality of life	“If I’m already in a declining state of health, I think that my inclination would be to not bother. Because I value quality of life. And I know that once you start on the path of cancer treatment, it is not a pretty path.” (Participant 24)
	“At my age, what I want to do is just…it sounds silly, but it comes with some age. I just want to get on with my life. Remove the cancer, let’s monitor me for a while and let me get on with my life with the least amount of invasive procedures so that I can maintain what quality of life I have right now. I know that sounds weird, but it does change as you, as I got older. I wouldn’t be saying this if I was 40, but I’m 70.” (Participant 25)
Family	“It’s interesting that one of the things that I have found is cancer is probably one of the diseases that is not just an individual disease, it affects your family and the people around you. So decisions are not just made by the person that’s dealing with cancer. And I see that more and more that you have a support system that you need to rely on for a disease like this. Some medical problems like an appendectomy are pretty simply solved. This is not one of those situations.” (Participant 10)
Unique life circumstances	“I think because of my age that I am certainly not ready to have the end of my life looking at me. But on the other hand…I don’t want to add to it and go to any length of finding out more and going through, putting my family through it. I have a husband, unfortunately, that has the start of dementia, so I’m his caregiver and I think I need to be healthy enough to take care of him and not put him through that sort of thing.” (Participant 13)
	“I think for older folks, if they’re married, what’s the condition of their spouse? Condition of children?” (Participant 17)

## Discussion

Current guidelines recommend against routine axillary staging with SLNB and postlumpectomy radiotherapy in women aged 70 years or older with early-stage, HR-positive breast cancer.^7,24^ However, both are used at high rates nationally, suggesting that there are reservations about these recommendations from clinicians, patients, or both.^10,11,12,13,25,26^ Our study found that, although most older women accepted the premise of age-based treatment strategies, there were several competing factors, including current health status, threats to quality of life, need for patient autonomy, and uncertainty about research informing current guidelines, that would influence their decision-making if they received a diagnosis of breast cancer. These findings are consistent with those of other studies^14,27,28,29,30^ that have shown that although many patients are wary of the motivations underlying efforts to reduce low-value services, older adults are generally supportive of avoiding overtreatment if clinicians actively engage patients in decision-making.

A key emergent theme is the importance of ensuring that older women are correctly interpreting their favorable breast cancer–specific prognosis. Participants frequently justified low-value treatments because of concerns for disease progression and death if they did not pursue aggressive action. In contrast, participants who accepted the expected good prognosis in the presented scenarios expressed comfort with treatment omission. Because older women may be more accustomed to conversations around end-of-life care in the context of cancer treatment, it could be counterintuitive to learn that recommendations for omission of certain therapies is based on a good, rather than poor, prognosis. This suggests that emphasizing the high likelihood of a positive breast cancer–specific prognosis could reassure older patients that avoiding overtreatment will reduce harms while providing equivalent benefit.

This approach relies on communicating statistical information and accurately presenting risks of various outcomes to patients. In our study, many participants claimed they were likely healthier than average and more likely to benefit from aggressive treatments. This feeling of personal exceptionalism and desire to pursue low-value services is likely associated with a tendency for patients and clinicians alike to overestimate the benefits and underestimate the risks of medical treatment.^31,32,33^ For example, a study^13^ of surgeons and radiation oncologists found that physicians overestimate patients’ life expectancies, incorrectly attribute radiotherapy to an improvement in survival rather than an improvement in local recurrence, and overestimate the risk of recurrence with radiotherapy omission in older women. One strategy to improve accurate risk communication is to optimize the format in which clinicians provide estimations.^34^ For example, although clinicians traditionally communicate risk either numerically or verbally, studies have found that pictographs significantly improve the ability to comprehend incremental risk reduction.^35^

Similarly, positively reframing these guidelines may address women’s concerns about age discrimination. Some women asserted that older patients were unfairly receiving less medical care but did not fully understand the motivations underlying these recommendations, in this case a good rather than poor prognosis from breast cancer. Conversely, other women thought age-based guidelines actually supported their autonomy by providing them an option to omit treatment. At times, clinicians may worry that their ethical obligation to reduce costs and harms associated with the provision of low-value services conflicts with the ethical principle of patient autonomy.^36^ However, studies in behavioral economics demonstrate that patients often do not make choices that are logical, values-concordant, or in their best interest.^37^ Thus, there is a potential role for nudging or beneficent persuasion, which is distinct from manipulation because of the transparency involved.^38^ When performed ethically, persuasion can be used to direct patients to their priorities and motivate them toward a beneficial and values-concordant decision.^39^ Thus, focusing on values important to older women, such as quality of life and maintaining functional status potentially threatened by overtreatment, may help patients feel comfortable with treatment omission. For example, a study^28^ found that older patients are interested in engaging in conversations with their clinician about reducing low-value cancer screening, particularly when this decision is framed in a way that emphasizes the patient’s own health priorities.

Another key finding of this study is that reducing the use of SLNB and adjuvant radiotherapy may require separate strategies, because participants viewed the potential contributions of these treatments differently. SLNB was perceived as a low-risk prognostic test potentially providing peace of mind or informing future treatments. These perceptions are shared by clinicians; a qualitative study^12^ of surgeons found that the belief that SLNB influences downstream treatment decisions is a major barrier to decreasing SLNB rates. However, although studies have shown that nodal positivity is associated with more adjuvant therapy, this additional therapy does not increase breast cancer–specific survival.^40^ In fact, simulations that suggest older patients with HR-positive breast cancer may experience negative quality-adjusted life-years if they receive chemotherapy compared with endocrine therapy alone.^40,41^ Therefore, research-informed education of both clinicians and patients on the risks, costs, and potential downstream consequences of SLNB is necessary. The Figure shows a conceptual model illustrating potential clinician strategies in response to patient-level challenges to the de-escalation of low-value breast cancer therapies in older women.

**Figure.**
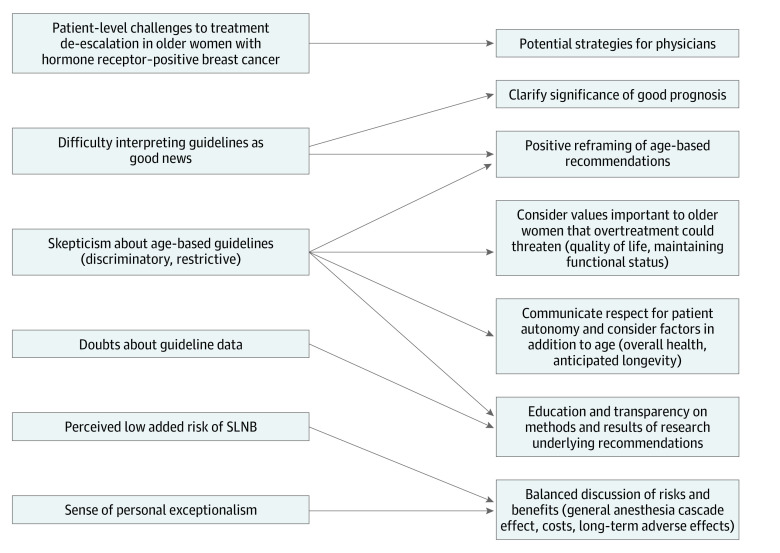
Conceptual Model of Potential Physician Strategies in Response to Patient-Level Challenges to the De-escalation of Low-Value Therapies in Women Aged 70 Years or Older With Early-Stage, Hormone Receptor–Positive Breast Cancer SLNB indicates sentinel lymph node biopsy.

In contrast to SLNB, most participants in our study would prefer to omit radiotherapy, viewing it as additive and unnecessary. Women frequently cited fears of radiotherapy and concerns about the burden of attending daily appointments for radiotherapy; this contrasts with the perceived convenience of SLNB, which is performed with resection of their primary tumor. These findings suggest that determinants at the clinician or institution level, rather than the patient level, likely contribute to high rates of postlumpectomy radiotherapy in older women. Efforts to decrease radiotherapy use may benefit from clinician-facing strategies balancing the desire for a modest improvement in locoregional recurrence rates with older women’s priorities, such as quality of life.

### Limitations

This study has several limitations. Although we purposefully sampled to include a diverse group of women, our recruitment method tended to select for White, highly educated, and high-functioning participants. We recognize that the views of this patient population may differ from those of other demographic groups. However, research^42^ on disparities in health care suggest that minority women and those of lower socioeconomic status are at risk of poorer outcomes and undertreatment for breast cancer. Conversely, a systematic review^43^ on overtreatment found that in circumstances in which race was a significant factor associated with the use of low-value services, nearly all studies suggested that White patients were more likely to receive unnecessary care. In addition, the incidence of breast cancer, particularly in older women, is highest in White women.^2^ Thus, we anticipate the patient population we have sampled may be at greater risk for overtreatment if diagnosed with breast cancer.

Furthermore, participants in this study were presented with hypothetical scenarios. Thus, it is possible that women would make different decisions when facing an actual diagnosis or when provided with a more extensive real-world medical consultation with more time to reflect on their decision. However, we think that the factors found to be associated with women’s decision-making, such as their interpretation of guidelines, views on aging, and secondhand experiences, are static constructs related to their personal values and history and are relevant in both real-life and theoretical scenarios.

## Conclusions

This qualitative study explored patient perspectives on low-value breast cancer treatment options for older women who receive a diagnosis of early-stage, HR-positive breast cancer. Positive reframing of recommendations to avoid SLNB and radiotherapy may be a strategy to reduce overtreatment while maintaining patient autonomy. Although patient preferences for SLNB could partially explain high rates of SLNB use nationally, persistent use of adjuvant radiotherapy may be associated with factors related to guidelines, health professionals, incentives or resources, and capacity for organizational change.

## Notes

Supplement. **eAppendix.** Phone Interview Guide for Breast Cancer Treatment Options in Older Patients
