# Supplementary material for: Patient Perspectives on Treatment Options for Older Women With Hormone Receptor–Positive Breast Cancer: A Qualitative Study
Source: JAMA Netw Open. 2020 Sep 22;3(9):e2017129. doi: 10.1001/jamanetworkopen.2020.17129 (PMC7509630; doi:10.1001/jamanetworkopen.2020.17129)
Supplement: Supplement. — eAppendix. Phone Interview Guide for Breast Cancer Treatment Options in Older Patients [file jamanetwopen-e2017129-s001.pdf]

Supplementary Online Content

Wang T, Mott N, Miller J, et al. Patient perspectives on treatment options for older women with hormone receptor–positive breast cancer: a qualitative study. *JAMA Netw Open*. 2020;3(9):e2017129. doi:10.1001/jamanetworkopen.2020.17129

eAppendix. Phone Interview Guide for Breast Cancer Treatment Options in Older Patients

This supplementary material has been provided by the authors to give readers additional information about their work.

© 2020 Wang T et al. *JAMA Network Open.*

eAppendix. Phone Interview Guide for Breast Cancer Treatment Options in Older Patients

Informed Consent (Verbal)

We are doing a study to better understand patients' opinions about treatment options for breast cancer. We expect it to take about 45 minutes to complete the interview.

Participating in the interview is voluntary. You don't have to participate if you'd rather not and you can stop at any time. You can skip any questions that you don't want to answer, whatever the reason, and you don't have to tell us why.

We will record this phone conversation. To keep your information confidential, we will transcribe the recording and remove any information that identifies you from the transcript. We'll destroy the recording when the study is finished. Although we'll keep a list of the people who participated, no one outside our study team will be able to figure out who participated or which people gave which answers. We plan to publish what we learn from this study, but we won't include any personal information that could reveal who answered the survey.

To thank you for taking part in our study, we'll send you a $25 gift card after you complete the interview. I'll ask for your name and mailing address so that I can send you the gift card.

Do you have any questions? Do you agree to participate in the study?

There are no right or wrong answers and feel free at any time to ask me to repeat or clarify anything.

Scenario 1:

Imagine you were recently diagnosed with breast cancer. Your doctor tells you that you have early stage cancer, meaning that the cancer is small, it doesn't appear to have spread, and the cancer is hormone positive. Based on this, your doctor states that you have the most common type of breast cancer in older women and have a good prognosis. The doctor recommends that you undergo a surgery to remove the cancer.

Does that scenario make sense? Do you have any questions?

1. Would you want a surgery to remove breast cancer? Why or why not?
    a. A lumpectomy is a surgery which only removes the tumor but sometimes must be followed by radiation. A mastectomy is a more aggressive surgery which removes the entire breast. Would you want a lumpectomy or a mastectomy? Tell me about your reasons why.
    b. Would you always want to treat your cancer or are there situations in which you would watch and wait? Tell me about your reasons why.

Let's return to the scenario. Your doctor also states that a common procedure done at the same time as the surgery to remove the cancer is something called a sentinel lymph node biopsy. With this procedure, a few lymph nodes are removed from your armpit in order to determine whether the cancer has spread. Lymph nodes are small structures that help filter harmful substances in the body. A benefit of the sentinel lymph node biopsy is that information from it can potentially help guide treatment decisions like whether to treat you with chemotherapy. It can also offer some information about your prognosis. However, the procedure requires you to come to the hospital prior to the surgery in order to have a tracer material injected to identify the sentinel lymph node and has some risks such as requiring general anesthesia, potentially causing pain, or damaging nerves in the armpit. The most recent guidelines state that for patients 70 years or older, sentinel lymph node biopsy does not improve survival rates for breast cancers like these that already have a good prognosis. Therefore, your doctor states that performing a sentinel lymph node biopsy is optional and up to you to decide.

Does the scenario make sense so far? Do you have any questions? Feel free to ask any time if you'd like me to repeat anything. Do you have any questions about this recommendation?

If participant asks why SLNB does not result in a survival benefit in older women:

© 2020 Wang T et al. *JAMA Network Open.*

We think it may be because after 70, there is less time for the cancer to recur and more women on average are dying of other causes. As a result, there is no average improvement in survival after cancers like these which already have a very good prognosis.

2. If you were undergoing surgery to remove cancer, would you also want a sentinel lymph node biopsy performed at the same time? Why or why not?
    a. How do the risks versus the benefits of the procedure factor into your decision?
    a. Would this procedure offer you peace of mind if it finds that your cancer has not spread? How important is that peace of mind to you?
    b. How much does it matter to you that the results of this procedure might guide further treatment recommendations such as chemotherapy?
    c. Does it matter that regardless of the treatment, your doctor expects that your prognosis from breast cancer will be good? Why or why not?
    b. How much would you base your decision on your doctor's recommendation or opinion?
    c. Does it matter to you that current guidelines state that in older patients there is no survival benefit to performing this procedure? Why or why not?
    d. Do you think it is reasonable that guidelines recommend different treatments for older patients compared to younger patients for the same disease? Why or why not?
    e. Does it matter to you that performing this procedure requires general anesthesia? Why or why not?
    f. Does it matter to you that in order to do the sentinel lymph node biopsy, you would need to come to the hospital early to have a tracer material injected in order to identify the sentinel lymph nodes? Why or why not?

Let's return to the scenario. Let's say that the sentinel lymph node biopsy was performed and found that the cancer has spread. Your doctor recommends treatment with hormone therapy but also offers treatment with chemotherapy if you want. Your doctor states that in older patients compared to younger patients, there are much higher risks associated with chemotherapy such as damage to your kidneys, low blood counts, or nerve damage. However, receiving chemotherapy could potentially increase your chance of survival.

3. Would you want treatment with chemotherapy? Why or why not?
    a. How do the risks versus the benefits of chemotherapy factor into your decision?
    b. How much would you base your decision on your doctor's recommendation or opinion?
    c. Do you know anyone who has undergone chemotherapy? If yes, does that influence your decision at all and how?
    d. For participants who wanted SLNB but did not want chemotherapy: Does your desire to omit chemotherapy change your decision about whether to have a sentinel lymph node biopsy or not? If so, why?

Scenario 2:

Now this is a different scenario. However, like in the first scenario, imagine you were recently diagnosed with breast cancer. Your doctor again tells you that you have early stage cancer with a good prognosis. This time, you make the decision to undergo a lumpectomy. Your doctor tells you that most women would receive radiation after a lumpectomy to decrease the risk of the cancer recurring. However, new guidelines state that older women over age 70 with low-risk breast cancer do not need radiation. For most in this age group, radiation does not decrease the risk of the cancer coming back and does not increase survival. Therefore, your doctor states that radiation is optional and up to you to decide. Although radiation is usually well-tolerated in most patients, it does require patients to go to a radiation center daily for several weeks. Regardless of whether you decide to undergo radiation therapy, your doctor recommends treatment with hormone therapy, which requires taking a daily pill.

4. Would you want treatment with radiation? Why or why not?
    a. How do the risks versus the benefits of radiation factor into your decision?
    b. How much would you base your decision on your doctor's recommendation or opinion?
    c. Does it matter to you that current guidelines state that in older patients there is no survival benefit to giving radiation? Why or why not?

© 2020 Wang T et al. *JAMA Network Open.*

d. In this scenario, do you think it is reasonable that guidelines recommend different treatments for older patients compared to younger patients for the same disease? Why or why not?
e. How much does needing to go to a radiation center daily factor into your decision?
f. Do you know anyone who has undergone radiation? Does that influence your decision at all?
5. Would you be willing to take hormone therapy (a pill) every day for several years? There are some side effects associated with this medication as well such as hot flashes, fatigue, and blood clots. Why or why not?

Now let's move away from the scenarios and talk generally about some of the factors that would influence how aggressively you would treat a diagnosis of breast cancer.

6. Do you know anyone who has ever been diagnosed or treated for breast cancer?
    a. If yes, how does their experience influence your own opinions about breast cancer treatment?
7. How much would your current general health status influence how much treatment you wanted?
    a. Does that depend on age? Why or why not?
    b. What about other medical conditions? Why or why not?
8. Sometimes providers recommend less aggressive treatment for older patients if the prognosis is good. What are your thoughts on that?
9. What are your thoughts on using tests of an older patient's functional status to determine if a patient is a good candidate for more aggressive or less aggressive treatment? For example, what if we tested your balance, memory, and overall health and used that to base your cancer treatment decisions? There is some research that suggests that these tests can help predict a patient's life expectancy and risk of decline with some cancer treatments like chemotherapy.
10. How involved are you in your own medical decision-making?
    a. Do you tend to make your own decisions about your health or do you rely on trusting doctors?
11. If you did not choose to have a treatment that your doctor offered you and your cancer returned or progressed, would you regret not pursuing more aggressive treatment?
12. How much of your medical decisions are made from concern that you may later regret not doing something?
13. Have your thoughts about how aggressive to be evolved over time as you've aged?
    a. For participants who think less aggressive treatment for older women is reasonable: Some women we've heard from think that it's not reasonable because people are living longer lives these days, or because they believe it's very individual and so guidelines shouldn't be age-based. What do you think about that?
    b. For participants who think less aggressive treatment for older women is unreasonable: Some women we've heard from think it's probably reasonable because older bodies might respond differently to treatments, or because younger women might be more likely to live longer or have more family responsibilities. What do you think about that?

For these next questions, I am going to read a statement to you. Please tell me which response you agree with.

14. Sometimes medical action is clearly necessary, and sometimes it is clearly NOT necessary. Other times, reasonable people differ in their beliefs about whether medical action is needed. In situations where it's not clear, do you tend to lean towards taking action or do you lean towards waiting and seeing if action is needed? Importantly, there is no "right" way to be.

    Please answer on the 1-6 scale below:
    - 1 (I strongly lean towards waiting and seeing)
    - 2 (I lean towards waiting and seeing)
    - 3 (I somewhat lean towards waiting and seeing)
    - 4 (I somewhat lean towards taking action)
    - 5 (I lean towards taking action)
    - 6 (I strongly lean towards taking action)

15. More medical treatment is usually better
    - Strongly agree

- Somewhat agree
- Neither agree nor disagree
- Somewhat disagree
- Strongly disagree

16. Providers often recommend services patients do not need
    - Strongly agree
    - Somewhat agree
    - Somewhat disagree
    - Strongly disagree

17. My own provider often recommends services I do not need
    - Strongly agree
    - Somewhat agree
    - Somewhat disagree
    - Strongly disagree

Would it be ok for me to ask a few questions about you for demographic purposes?

18. How old are you today?
19. What zip code do you live in?
20. What is your race/ethnicity?
21. What is your highest education level?

That's the end of the interview questions. Thank you very much. Do you have anything you'd like to add?

© 2020 Wang T et al. *JAMA Network Open.*
